# Evaluation of anterior fixed appliances on maxillary arch growth in preschool children

**DOI:** 10.1038/s41598-025-87005-8

**Published:** 2025-01-29

**Authors:** Mohamed Ghaly, Nahed Abo Hamila

**Affiliations:** Faculty of Dentistry, Tanta University, Tanta, Egypt

**Keywords:** Fixed, Nance, Growth, Paediatric dentistry, Dental trauma

## Abstract

This study aimed to evaluate a new modified fixed appliance for rehabilitation of premature loss of anterior teeth in preschool children versus a modified Nance appliance on maxillary arch growth with parental satisfaction. The study was conducted as a clinical trial and it was carried out at Pediatric Dentistry Department, Faculty of Dentistry, Tanta University. Forty preschool children from both genders aged from 3–5 years were included in the study. The selected children were divided into two groups; Group I: Twenty children received a modified fixed bridge. Group II: Twenty received a modified Nance appliance. Evaluation of both appliances on maxillary growth was carried out at baseline, 6 and 12 months. Finally, parental satisfaction was also recorded. It was revealed that, there was continuous maxillary growth in both groups at different follow up periods with no sexual dimorphism, and parental satisfaction was significantly higher in group I than group II. A modified fixed bridge was better than Nance appliance regarding parental satisfaction and it doesn’t interfere with maxillary growth.

## Introduction

Premature loss of primary anterior teeth in preschool children is often attributed to fall down accidents, trauma or advanced decay of teeth^1^. Parents are first that have great concern about unpleasant aesthetics of their children due to loss of these teeth. Moreover, there are several sequelae arising from premature loss of primary incisors such as improper quality of life, difficult mastication, speech problems, loss of arch integrity, interference with proper development and eruption of succedaneous teeth as well as development of abnormal oral habits^2^.

On the other hand, William et al., 2001 observed that such sequelae were not necessary to occur as they hypothesized that mastication may not be affected, problems of speech are not common and if occurred they are often compensated and self-corrected and space loss are very rare. They suggested that the esthetic is considered only reason to persuade the parents to restore anterior extraction situation of their preschool children with proper appliance^3^.

Even so, multiple attempts were carried out to compensate premature loss of anterior teeth for preschool children, such as removable appliance but it did not receive acceptance by children and their parents because it requires more cooperation of child, also its controversial effect on transverse growth of the dental arch, finally frequent inflammation of soft tissues and periodontium^4^.

Subsequently, fixed appliance was preferred than removable one as it was approximately free from such drawbacks. Groper etal,0.1984 introduced fixed appliance for rehabilitation of premature missing of primary incisors in preschool children, the Groper appliance consisted of two metal bands around second primary molars with wire extends from them toward anterior edentulous area where acrylic teeth are attached to metal cleats that are soldered to the palatal wire bar, but it’s durability was questionable ^5^ .

Moreover, Hollywood bridge (modified Nance appliance) was developed later, it was similar to Groper appliance but acrylic teeth were secured into palatal acrylic button in rugae area. Since there was minimal palatal coverage, it was more tolerable by children. However, acrylic portion may irritate underlying soft tissues and sometimes damage to anchoring teeth such as root resorption may occur^6^.

According to these points a new modified fixed appliance was developed and evaluated regarding maxillary arch growth and parental satisfaction.

## Methods and materials

### Study design

A randomized clinical trial design was adopted in this present research.

### The study setting

Clinical procedures were performed at Pediatric Dentistry Department, Tanta University.

### Sample size calculation

The sample size was calculated by using Epi-Info computer software version 7, assuming that confidence level at 95% with 5% margin of error and design defect in power analysis in two calculated samples was 20 patients.

### Ethical considerations

The purpose of this study was explained to the parents and they were informed that all prepared abutment teeth would be compensated by single crowns after removal of the bridge to protect them until exfoliation time then written informed consents were obtained from the parents according to the guidelines on human research performed by the Internal Research Ethics Committee, Faculty of Dentistry, Tanta University (info_REC@dent.tanta.edu.eg).

All methods were performed in accordance with the relevant guidelines and regulations.

### Approval of the trial protocol

The trial protocol was approved by the Internal Research Ethics Committee, Faculty of Dentistry, Tanta University in 2/11/2022.

### Study sample and group assignment

**Fig.1:**
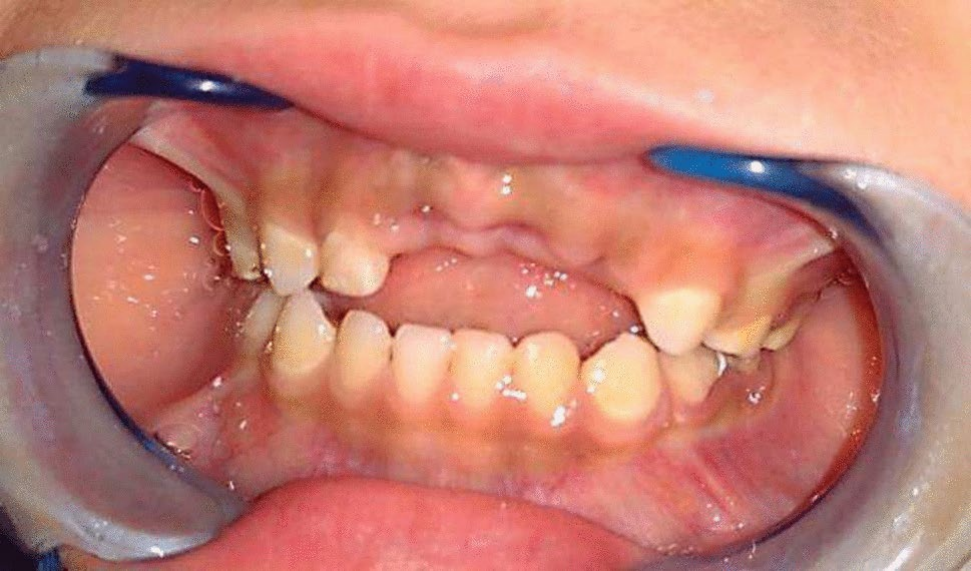
Premature loss of upper ant teeth.

**Fig.2:**
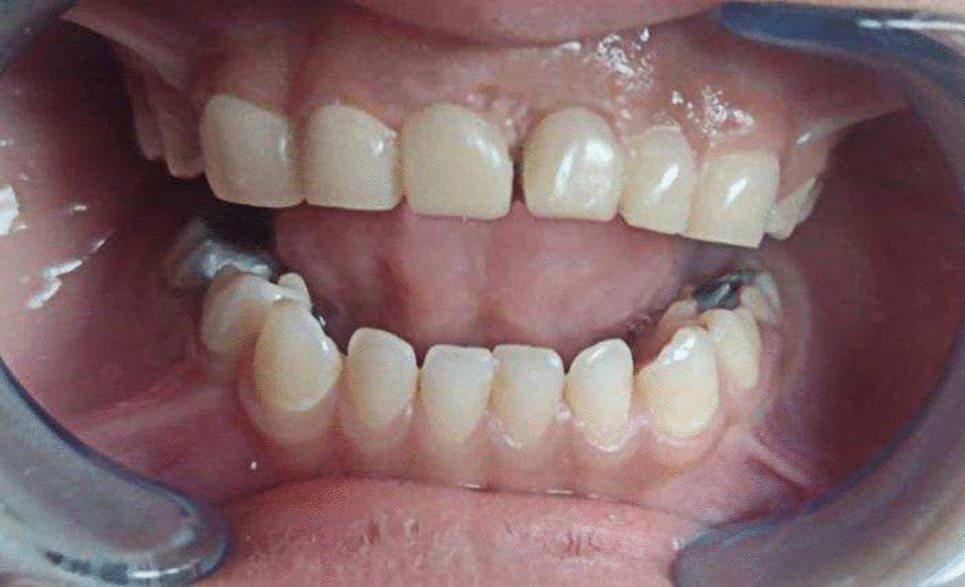
Rehabilitation of extraction site with modified fixed bridge.

**Fig.3:**
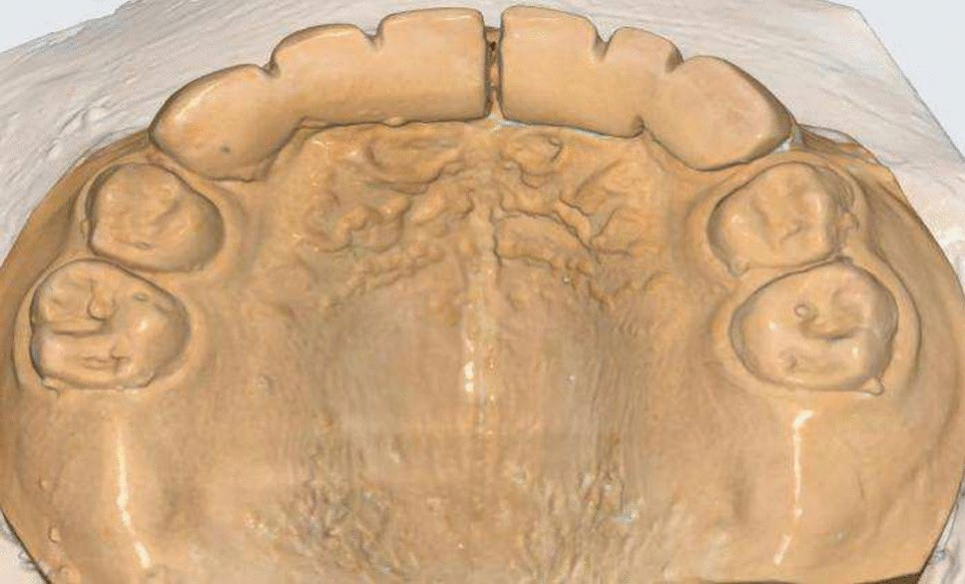
Palatal view of modified fixed bridge.

A total of forty preschool children from both genders aged between 3–5 years old were selected and randomly assigned (Through closed envelopes) into two groups as follow:

Group I (study group): Twenty preschool children used a modified fixed bridge in their anterior extraction site. (Fig1, 2, 3)

**Fig.4:**
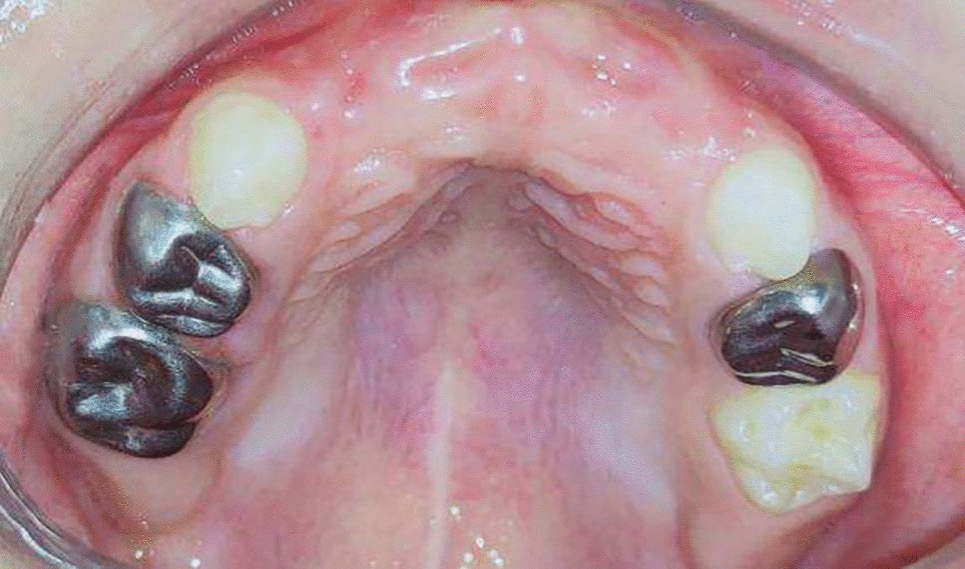
Premature loss of upper ant teeth.

**Fig.5:**
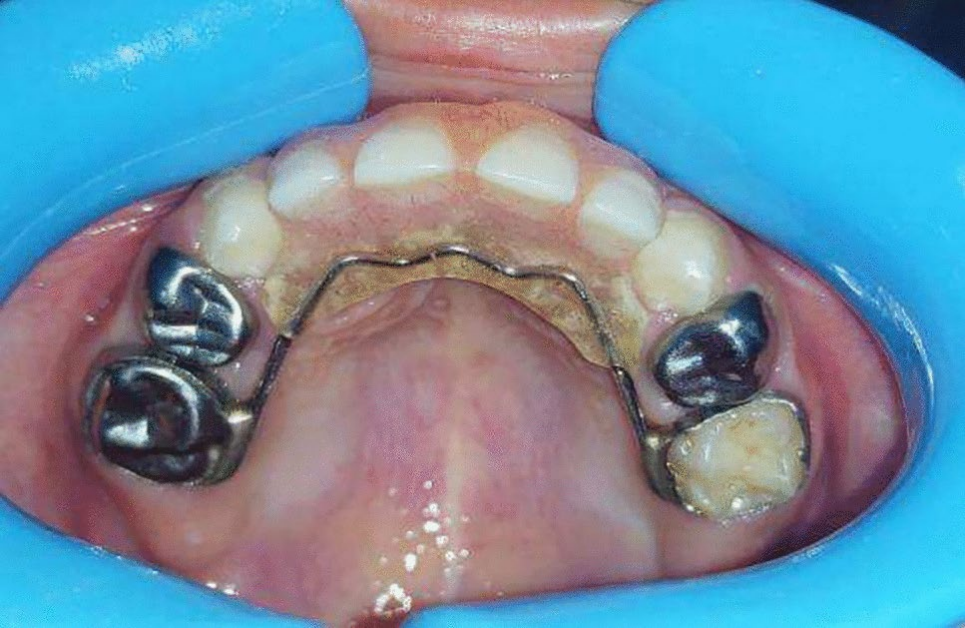
Rehabilitation of extraction site with modified Nance appliance.

**Group II** (control group): Twenty preschool children used a modified Nance appliance in their anterior extraction site. (Fig4, 5)

- **Inclusion criteria**^6^**:**
- Healthy cooperative children
- **Children with normal occlusion**
- Sound abutment teeth **(Primary canines)**
- Premature loss of primary incisors due to trauma or damaged stage of rampant caries
- children from both genders aged between 3–5 years old were selected
- **Exclusion criteria**^7^**:**
- Medically compromised children or children with special needs.
- Permanent teeth were near to eruption as evaluated in x-ray film.
- Badly destructed abutment teeth or exhibit root resorption as appeared in preoperative x-ray.
- Children with bruxism or abnormal habits.
- **Children with abnormal occlusion like cross-bite or deep-bite**

Practical procedures

### Group I

Each child was examined carefully to be compatible with inclusion criteria then preoperative preapical x-ray film was captured to assess stage of succedaneous teeth development, ensured that there were not any root fragments of primary incisors and evaluated the roots of abutment teeth.

**Fig.6:**
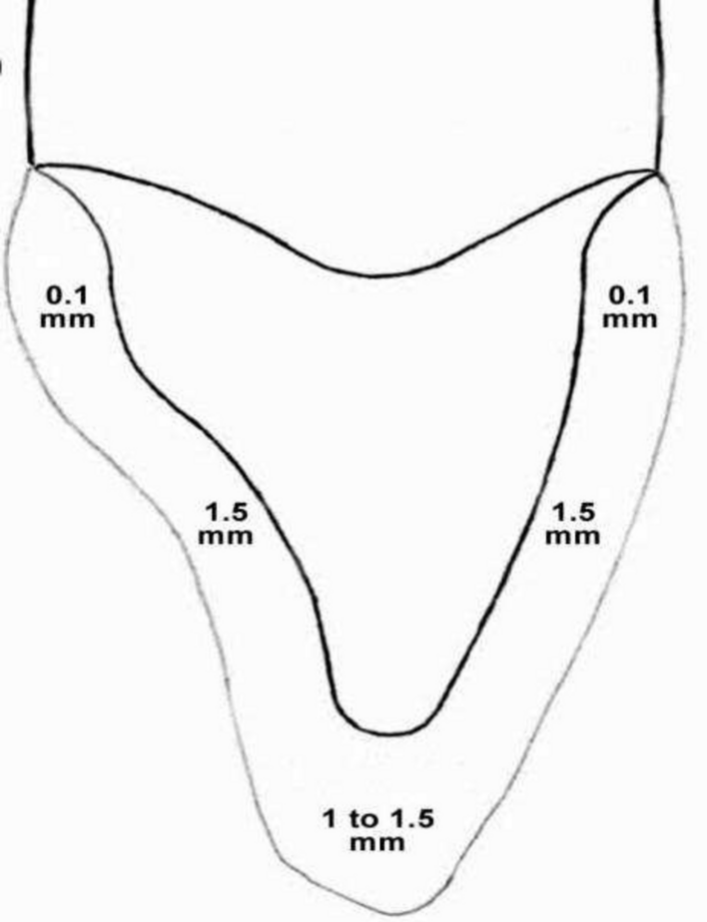
Schematic figure of the prepared tooth.

Topical anesthesia was applied before administration of local anesthesia for abutment teeth before preparation. Then preparation of abutment teeth was carried out according to manufacturing instructions for acrylic fixed bridge (Polymethyl methacrylate acrylic (PMMA)) by using tapered diamond bur (Komet® 8862.FG.010, Feather Edge Diamond Preparation Bur, Germany). As conservative preparation was followed, first incisal reduction (1–1,5mm) was performed to obtain incisal clearance then vertical grooves was applied on labial and lingual surfaces by feather edge diamond bur to guide the amount of the required reduction then follow uniform and anatomical reduction in all surfaces while tapered diamond bur was parallel to long axis of the abutment tooth, consequently reduction with slight incisal convergence was obtained. Finally smoothness of all sharp edges was performed. (Fig. 6).

Impression with putty rubber base material was taken (Zhermack Zetaplus Putty Impression Material, Germany) according to manufacturing instructions as was placed into an impression tray; then they were both inserted into child mouth and pushed onto their teeth in order to take an impression. Once the dental putty was set the impression was removed. An impression was also taken of the opposing teeth, so the technician could see how child bite together. A temporary crown (Charm Temporary Crown^R^ ) was cemented onto the prepared tooth by (Charm Temporary cement) to protect them until the bridge was being fabricated. The impressions were sent to the dental laboratory.

**Fig.7:**
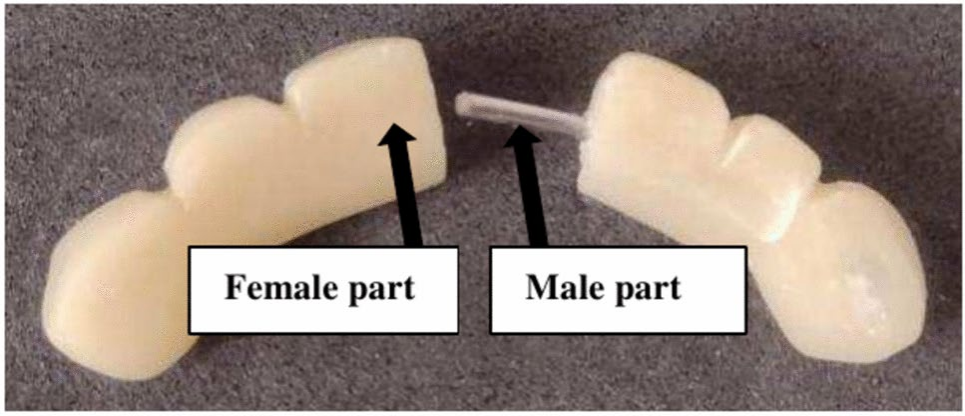
New Modified fixed bridge.

**Fig.8:**
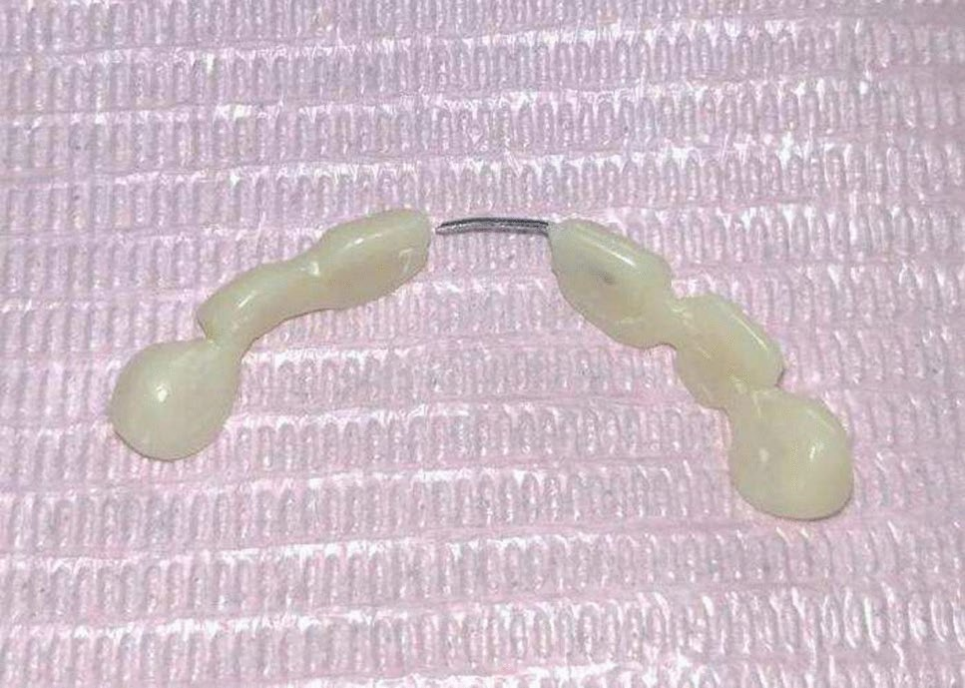
New Modified fixed bridge.

A modification was carried out in the bridge by split it through midline into two segments, then 1cm of stainless steel wire with diameter 0.8 mm was embedded into one segment (Male part) with free end, while the other segment a small tunnel was prepared to allow insertion of free end of the stainless steel wire into it (Female part) (Figs. 7, 8).

After fabrication of the modified bridge, it was received from the lab then temporary crowns were removed and the prepared abutment teeth were totally cleaned from any cement. Finally, the appliance was cemented with conventional glass ionomer cement.

### Group II:

The first step was done in group (I) was performed, then primary impression was taken for selection appropriate stainless-steel bands on second primary molars. After proper adaptation of the bands, secondary impression was taken and sent them to the dental laboratory for construction of modified Nance appliance.

A modified Nance appliance was constructed by 0.9 mm rigid stainless steel wire welded to each band then extended to the raugae area of hard palate where heat cure acrylic resin poured into it, then part of the acrylic resin extended into alveolar ridge as artificial acrylic teeth would embedded in the acrylic resin. Figure 5

Evaluations of maxillary development for both groups were assessed by measuring the inter-canine arch width as a parameter of anterior maxillary growth immediately (base line) after insertion of appliances, at six and twelve months follow up. Also, parental satisfaction about performance of each appliance was evaluated at the end of the study by questionnaire similar to one used by Kupietzky and Waggoner^8^.

The questionnaire was mentored for ease of understanding on forty parents who attended the pediatric dentistry clinics Faculty of Dentistry, Tanta University. A trained dental practitioner explained the questionnaire to the accompanying parent. Also treated children were not present during the interview time. The parents evaluated their child’s restoration directly.

### Measurement of inter-canine arch width was carried by Digital vernier caliper

**Fig. 9:**
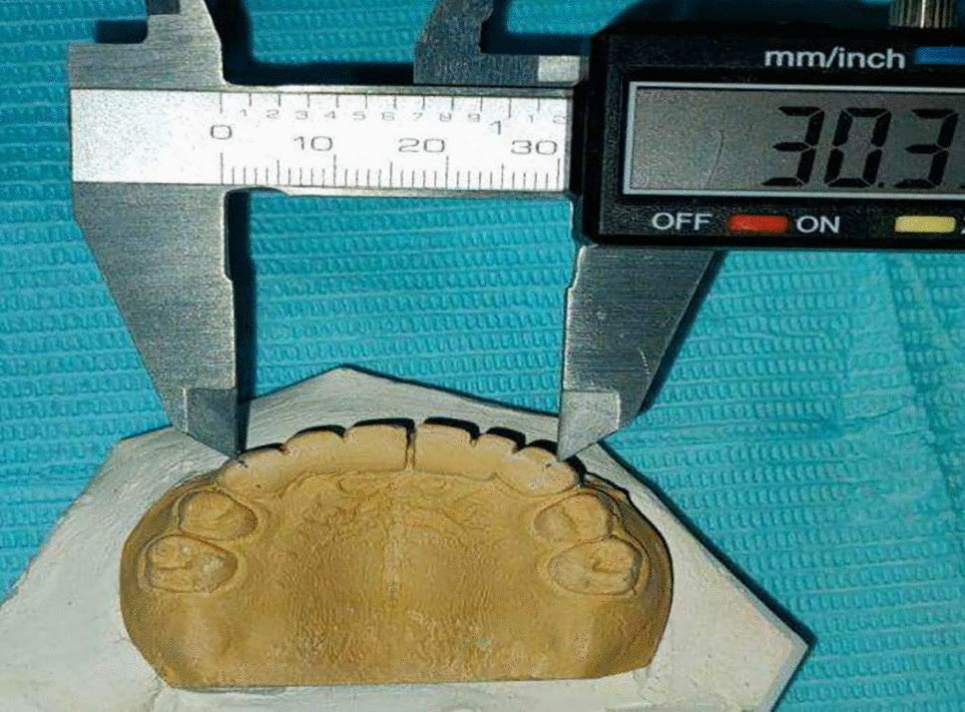
Measurement of inter-canine arch width by Digital caliper.

- First, dental casts that were obtained by taking an impression after insertion of appliances immediately, six and twelve months follow up.
- Marking of reference points on the dental casts were performed by a sharp lead pencil on cusp tips of each upper primary canine, so inter-canine arch width could be measured as distance between two cusp tips by Digital vernier caliper (Zurcher model 042,751, Dentaurum, GmbH & Co., Ispringen, Germany). Figure 9.

- A single examiner was responsible for performing all measurements to avoid any intra-observer error. The intra-examiner repeatability of the measurements was determined to be 0, 2 or less.
- To evaluate any error in identification of the landmark, five dental casts were randomly selected and measured twice by the same examiner over period of one week.

### Data analysis

Shapiro–Wilk test used to test the normal distribution of the data. Statistical analysis of all data were done using SPSS 24 (IBM, Armonk, NY, United States of America) after they were collected and tabulated. Independent-samples t-test was used when comparing between two groups. Paired t-test was used when comparing between two times in same group. Chi-square (X2) test was used in order to compare proportions between two qualitative parameters.

## Results

**Fig. 10:**
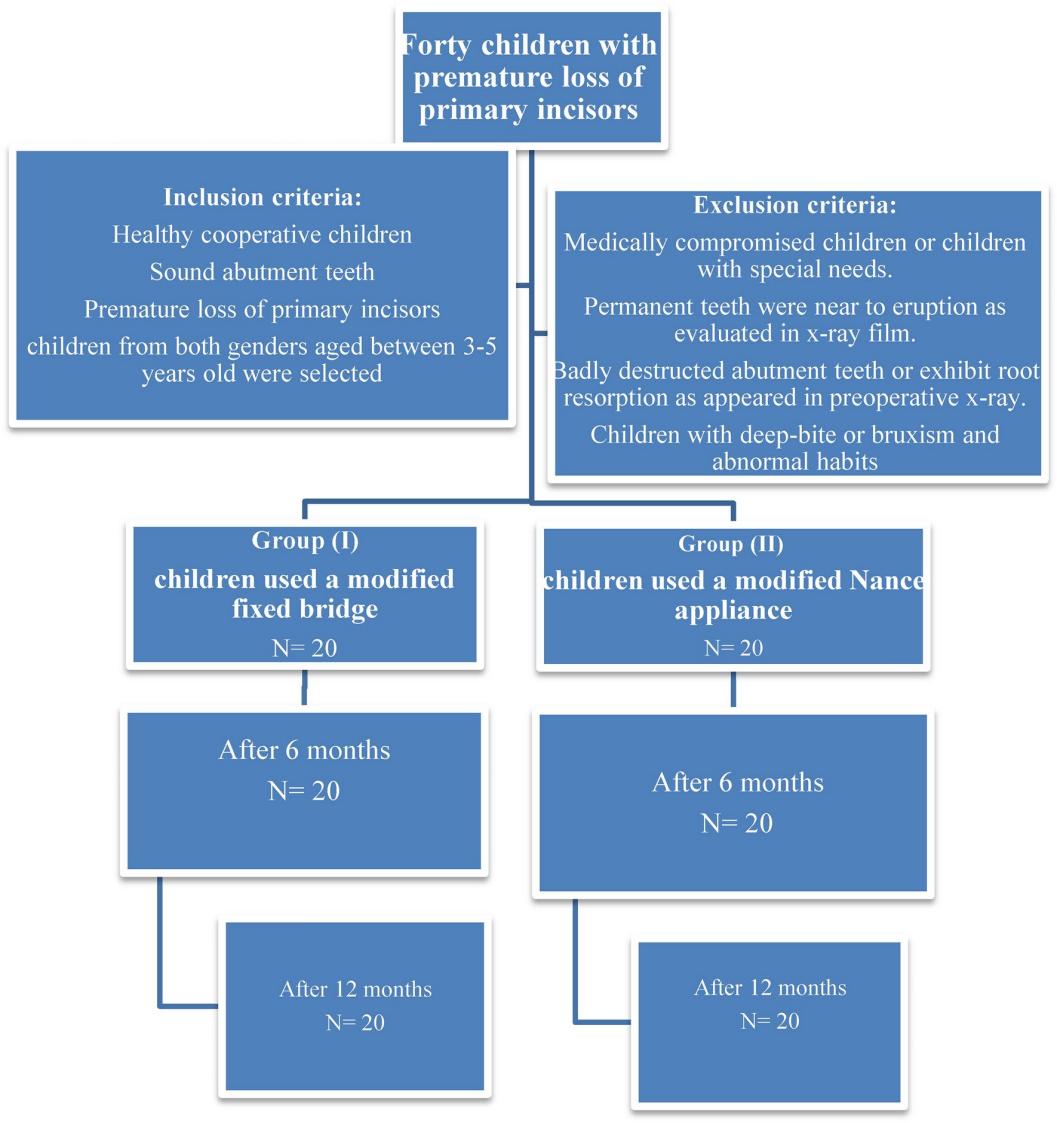
Measurement of inter-canine arch width by Digital caliper.

The current study was conducted on forty preschool children. Clinical evaluation of both appliances was done at different follow up period with no complain of both them. However, there were two cases with a modified Nance appliance showed fractured of one acrylic tooth, and it was repaired easily. Measurement of inter-canine arch width was performed by digital caliper where level of accuracy was appropriate. (Illustrated in patient flow chart Fig. 10).

### In relation to measurement of inter-canine arch widths for both genders

The results of inter-canine arch width for males at different follow up periods in group I illustrated that the mean of inter-canine arch width were 27.60 ± 0.90, 27.98 ± 1.04, 28.42 ± 0.97 at baseline, six months and twelve months respectively. While the mean of inter-canine arch width for females were 27.58 ± 1.02**,** 28.02 ± 0.99**,** 28.41 ± 1.05 at baseline, six months and twelve months respectively. There was no statistically significant difference between the mean of both males and females in group I (p > 0.05).

Similarly in group II, there were no statistically significant differences between the mean of both males and females at different follow up periods as (p > 0.05). As shown in Table.1

**Table 1:** Comparsion between females and males for inter-canine arch widths in both groups

			Male			Female			t. test	p. value
Group I	Base line	Range	26.35	–	29.41	26.32	–	29.32	0.051	0.960
		Mean ± SD	27.60	±	0.90	27.58	±	1.02		
	6 m	Range	26.84	–	30.12	26.85	–	29.74	0.070	0.945
		Mean ± SD	27.98	±	1.04	28.02	±	0.99		
	12 m.	Range	27.32	–	30.45	27.12	–	30.21	0.013	0.990
		Mean ± SD	28.42	±	0.97	28.41	±	1.05		
Group II	Base line	Range	26.78	–	29.21	26.69	–	29.14	0.761	0.456
		Mean ± SD	27.87	±	0.82	27.59	±	0.78		
	6 m	Range	27.12	–	30.05	26.97	–	29.54	0.826	0.420
		Mean ± SD	28.30	±	0.90	27.99	±	0.76		
	12 m.	Range	27.35	–	30.32	27.12	–	29.75	0.875	0.393
		Mean ± SD	28.81	±	0.98	28.47	±	0.72		

### Comparison between group I and group II for inter-canine arch widths at different follow up periods was illustrated in Table 2:

**Table 2:** Comparsion between group I and group II for inter-canine arch widths

Group I					Group II			t. test	p. value
Base line	Range	26.32	–	29.41	26.69	–	29.21	0.520	0.606
	Mean ± SD	27.59	±	0.93	27.73	±	0.79		
6 m	Range	26.84	–	30.12	26.97	–	30.05	0.512	0.612
	Mean ± SD	28.00	±	0.99	28.15	±	0.82		
12 m.	Range	27.12	–	30.45	27.12	–	30.32	0.774	0.444
	Mean ± SD	28.41	±	0.98	28.64	±	0.85		
Base line & 6 m		0.004*			0.003*				
Base line & 12 m		0.001*			0.001*				
6 m & 12 m		0.001*			0.001*				

The results showed that there were no statistically significant differences between the mean of inter-canine arch width of both groups at baseline, six months and twelve months respectively (p > 0.05). On other hand there was statistically significant difference between the mean of inter-canine arch width at different follow up periods in the same group (p < 0.05). As shown in Table.2

### Parental satisfaction

The results showed that there was a statistically significant difference between group I and group II in relation to shape and overall satisfaction (p < 0.05). Regarding to durability, the results illustrated that there was no statistically significant difference between both groups (p > 0.05). As shown in Table 3

**Table 3:** Comparsion between group I and group II for parental satisfaction

		Group I		Group II		X2	P value
		N	%	N	%		
Shape	Satisfied	18	90	8	40	10.988	0.001*
	Dissatisfied0	2	10	12	60		
Durability	Satisfied	17	85	16	80	0.172	0.677
	Dissatisfied	3	15	4	20		
Overall satisfaction	Satisfied	18	90	9	45	9.231	0.002*
	Dissatisfied	2	10	11	55		

## Discussion

Premature loss of anterior primary teeth is considered critical issue that requires proper intervention as it affects mainly esthetic, speech and quality of life of children^9^. Likewise, a study was conducted by Sharma et al., 2019, they concluded that parental desire, restoration of the esthetic, maintaining of the space and function are the main reasons for replacement of upper anterior edentulous area with an esthetic appliance. They also found that in appropriate speech might be occurred especially sounds such as "s", "z" and “th” were mainly affected ^10^.

However, some authors recommended that premature loss of primary incisors is often given little clinical attention unless severe space closure is observed or there is evidence of critical speech problem and developing of abnormal oral habits especially tongue thrust habit with sequelae of dental and skeletal malocclusion^1,11,12^.

In the current study, a modified fixed bridge was used versus a modified Nance appliance for rehabilitation of upper anterior extraction area for both males and females’ children due to different growth rates between them. Both appliances were evaluated on the inter-canine arch widths of maxillary arch which a one parameter of anterior maxillary growth by directly and conventional method with digital caliper as accurate measurements could be obtained after inter-examiner repeatability.

Concerning to inter-canine arch width measurements (ICW), there was no a statistically significant differences between the mean of both males and females in group I and group II at different follow up period, this was consistent with Shih et al., 2016, who documented that there was no statistically significant difference between the genders for their ICW after replacement of anterior extraction site with kiddy denture for preschool children^13^.

These results were also agreed with study conducted by Ciusa et al. 2007^14^who found that no sexual dimorphism for all ICW values in Italian children between the ages of 3 years and 6 years. On the contrary, a study conducted by Bishara et al. 1997^15^, showed that there was sexual dimorphism for ICW values for children aged from 3 to 5 years, where males were found to have a 0.5 mm greater increase in ICW than females.

As regarding to continuation of maxillary growth during follow up period of both appliances, Table V.2 explained that the values of ICW were increased by about 0.5 mm after six months and about 0.9 mm after twelve months respectively. Thus, there was no interference of both appliances on maxillary growth of preschool children.

These results were agreed with Ciusa et al. 2007^15^ who documented that ICW increased by 0.9 mm during the period from 3 years of age to 6 years of age with no sexual dimorphism. Likewise, Shih et al., 2016^14^, concluded that the amount of continuous maxillary growth was about 0.6 mm during the first 6 months after insertion of the kiddy dentures for preschool children and was about 1 mm during the 1st year.

In relation to parental satisfaction for both appliances, Parents were asked to rate parameters such as durability, shape and overall satisfaction. It was found the modified fixed bridge got a great acceptance by the parents than a modified Nance appliance and this due to acrylic flange of a modified Nance appliance that affects aesthetic of their children. Furthermore, acrylic button that rests on the rugae area could irritate soft tissues by times if there was no proper oral hygiene applied.

These results were consistent with Biedma-Perea etal.2023^16^ , who suggested that modified Nance appliance had some complications such as mucosal hyperplasia secondary to friction of the appliance, development of dental caries to neighboring teeth, failure of band cementaion and damage to anchorage teeth as resorption of their roots may occur.

On the other hand, Khare, et al. 2013, suggested that a modified Nance appliance revealed good success rate with improvement of the aesthetic and function of young children with little tissue irritation^17^.

Regarding to the durability of both appliances, although two cases showed fractured of one of resin teeth of the modified Nance appliance, there was no significant difference between them as it could be repaired easily, so it did not represent source of concern to the parents. This was agreed with Volpato et al., 2021, who clarified that the fracture of the resin teeth of the modified Nance appliance was attributed to excessive flexibility and lack of support of the appliance^18^.

The limitation of this study was: Fabrication of a modified fixed bridge appliance on two abutment teeth only was less retentive. Also we recommended increasing the period of the study more than one year.

## Conclusion

1. A modified fixed bridge appliance and a modified Nance appliance could restore of premature loss of anterior teeth in preschool children properly.
2. A modified fixed bridge appliance was preferred than a modified Nance appliance for rehabilitation of premature loss of anterior teeth in preschool children.
3. Both appliances don’t interfere with maxillary arch growth and a modified fixed bridge achieves high parental satisfaction regarding aesthetic and durability.

## Supplementary Information

Supplementary Information.

## Data Availability

All data generated or analyzed during this study are included in this published article [and its supplementary information files].
