# Supplementary material for: Evaluation of anterior fixed appliances on maxillary arch growth in preschool children
Source: Sci Rep. 2025 Jan 29;15:3628. doi: 10.1038/s41598-025-87005-8 (PMC11779851; doi:10.1038/s41598-025-87005-8)
Supplement: Supplementary file 1 — Supplementary Information. [file 41598_2025_87005_MOESM1_ESM.pdf]

Evaluation of Anterior Fixed Appliances on Maxillary Arch Growth in Preschool Children

***Mohamed Ghaly* [*], *Nahed Abo Hamila* [**]**

[*] *Lecturer of pediatric Dentistry, Faculty of Dentistry, Tanta University*

ORCID: *0000-0003-4500-2184*

Email: mohamed1ghaly@gmail.com

[**] *Professor of pediatric Dentistry, Faculty of Dentistry, Tanta University*

ORCID: *0009-0006-4824-4401*

Email: nahed_abohamila@yahoo.com

To Whom It May Concern:

RE: Evaluation of Anterior Fixed Appliances on Maxillary Arch Growth in Preschool Children

As project manager for the Pan African Clinical Trial Registry (pactr.samrc.ac.za) database, it is my pleasure to inform you that your application to our registry has been accepted. Your unique identification number for the registry is **PACTR202406653535866**

Clinical trial number:

PACTR202406653535866

Fund:

Authors have no fund

- **All data generated or analyzed during this study are included in this published article [and its supplementary information files]**
- **I had no documented consents for my participants**

The informed consent

موافقة المريض

اقر أنا

..

بانني اطلعت على بيان مفصل عن البحث المقدم من الطبيبة /

بقسم طب اسنان الاطفال و صحة الفم بكلية طب اسنان طنطا

واوافق طواعية على المشاركة في هذا البحث الذي يهدف الى :

وتم ابلاغي ايضا باحتمالية المتاعب التي يمكن حدوثها من الدراسة و هي

تحاط النتائج المترتبة على هذه الدراسة بالسرية التامة و لا تستخدم لاي غرض اخر غير البحث العلمي وقرات المعلومات السابقة وكانت لي الفرصة للسؤال عما اريد وتم الرد على اسئلتي كلها واوافق بكامل قناعتي على المشاركة في هذا البحث.

اسم الباحــث:

رقم التليفـون:

التوقيـــــع:

التاريـــــخ:

اسم الطفـــل:

ولي الامـــر:

رقم البطاقـة:

العنــــوان:

التليفـــون:

التوقيـــــع:

The questionnaire which was given to the parents

		Satisfy	Dissatisfy
**Shape**	**Aesthetic**		
**Durability**	**Serve its function without fracture**		
**Overall satisfaction**	**Convenient and meet your acceptance**		
